# 563 Self-management Interventions and Outcome Measures for Individuals with Chronic Traumatic Injury: A Scoping Review

**DOI:** 10.1093/jbcr/irae036.197

**Published:** 2024-04-17

**Authors:** Caitlin P Coates, Lauren Shepler, Mariana Velasquez-Cano, Colleen M Ryan, Jeffrey C Schneider, Lewis E Kazis

**Affiliations:** University of New England College of Osteopathic Medicine, Portland, ME; Spaulding Rehabilitation Hospital, Harvard Medical School, Boston, MA; Department of Physical Medicine and Rehabilitation, Harvard Medical School, Spaulding Rehabilitation Hospital, Charlestown, Massachusetts, Medellin, Antioquia; Massachusetts General Hospital/Shriners Children's, Boston, MA; Department of Health Law, Policy and Management, Boston University School of Public Health, Boston, MA; University of New England College of Osteopathic Medicine, Portland, ME; Spaulding Rehabilitation Hospital, Harvard Medical School, Boston, MA; Department of Physical Medicine and Rehabilitation, Harvard Medical School, Spaulding Rehabilitation Hospital, Charlestown, Massachusetts, Medellin, Antioquia; Massachusetts General Hospital/Shriners Children's, Boston, MA; Department of Health Law, Policy and Management, Boston University School of Public Health, Boston, MA; University of New England College of Osteopathic Medicine, Portland, ME; Spaulding Rehabilitation Hospital, Harvard Medical School, Boston, MA; Department of Physical Medicine and Rehabilitation, Harvard Medical School, Spaulding Rehabilitation Hospital, Charlestown, Massachusetts, Medellin, Antioquia; Massachusetts General Hospital/Shriners Children's, Boston, MA; Department of Health Law, Policy and Management, Boston University School of Public Health, Boston, MA; University of New England College of Osteopathic Medicine, Portland, ME; Spaulding Rehabilitation Hospital, Harvard Medical School, Boston, MA; Department of Physical Medicine and Rehabilitation, Harvard Medical School, Spaulding Rehabilitation Hospital, Charlestown, Massachusetts, Medellin, Antioquia; Massachusetts General Hospital/Shriners Children's, Boston, MA; Department of Health Law, Policy and Management, Boston University School of Public Health, Boston, MA; University of New England College of Osteopathic Medicine, Portland, ME; Spaulding Rehabilitation Hospital, Harvard Medical School, Boston, MA; Department of Physical Medicine and Rehabilitation, Harvard Medical School, Spaulding Rehabilitation Hospital, Charlestown, Massachusetts, Medellin, Antioquia; Massachusetts General Hospital/Shriners Children's, Boston, MA; Department of Health Law, Policy and Management, Boston University School of Public Health, Boston, MA; University of New England College of Osteopathic Medicine, Portland, ME; Spaulding Rehabilitation Hospital, Harvard Medical School, Boston, MA; Department of Physical Medicine and Rehabilitation, Harvard Medical School, Spaulding Rehabilitation Hospital, Charlestown, Massachusetts, Medellin, Antioquia; Massachusetts General Hospital/Shriners Children's, Boston, MA; Department of Health Law, Policy and Management, Boston University School of Public Health, Boston, MA

## Abstract

**Introduction:**

Self-management education programs have demonstrated efficacy in various chronic conditions to empower and educate patients to manage their treatment intervention options, work through disease related challenges, gain confidence about their treatment related decisions and ultimately improve their quality of life. Burns are increasingly recognized as a chronic condition, however there is limited data on self-management interventions in this population. Therefore, the purpose of this study is to perform a scoping review of self-management education programs for chronic traumatic injury populations to identify self-management interventions and gaps in knowledge in this population.

**Methods:**

Preferred Reporting Items for Systematic reviews and Meta-analyses guidelines were used to conduct a comprehensive search of peer-reviewed literature for articles from 2013-2023 using keywords relating to ‘self-management’ and ‘chronic traumatic injury’ in the following databases: PubMed (MEDLINE), Embase, Scopus, and ScienceDirect. Article inclusion criteria included: papers written in English, participants over the age of 18 years, studies focusing on physical traumatic injury populations. This scoping review is inclusive of studies focusing on participants of all races, genders, educational status, and employment status.

**Results:**

A total of 810 studies resulted from the initial database searches, resulting in 6 studies that met the inclusion criteria for the scoping review. Of the resulting literature 3 studies focused on individuals with Traumatic Brain Injury and 3 with Spinal Cord Injury. There were no studies in the burn population. Total sample size ranged from 7 to 84 participants in the included studies. Interventions were applied for varying study durations with a minimum of 21 days up to 586 days across the studies reviewed. There were 5 studies that utilized telephone calls to apply their intervention, making this the most common intervention modality. Notably of the studies reviewed, 5 studies showed a benefit to the self-management intervention applied meaning that they described an increase in either self-efficacy scores or overall participant satisfaction with the intervention.

**Conclusions:**

There are limited research studies examining self management interventions in the chronic traumatic injury population, thus identifying a gap in the knowledge surrounding self-management interventions in these populations. Of the six included studies, most demonstrated benefits with self-management interventions.

**Applicability of Research to Practice:**

This review establishes the need for scientific testing of a self-management intervention in the chronic traumatic injury population, inclusive of individuals living with a burn injury.

